# The impact of ultrasound-guided bilateral rectus sheath block in patients undergoing cytoreductive surgery combined with hyperthermic intraperitoneal chemotherapy — a retrospective study

**DOI:** 10.1186/s12871-020-01099-3

**Published:** 2020-08-11

**Authors:** Shaoheng Wang, Pengfei Liu, Teng Gao, Lei Guan, Tianzuo Li

**Affiliations:** Department of Anesthesiology, Beijing Shijitan Hospital Capital Medical University, Beijing, 10038 China

**Keywords:** Cytoreductive surgery, Hyperthermic intraperitoneal chemotherapy, Rectus sheath block, General anaesthesia, Analgesia

## Abstract

**Background:**

Rectus sheath block (RSB) is known to attenuate postoperative pain and reduce perioperative opioid consumption. Thus, a retrospective study was performed to examine the effects of bilateral rectus sheath block (BRSB) in cytoreductive surgery (CRS) combined with hyperthermic intraperitoneal chemotherapy (HIPEC).

**Methods:**

A total of 178 patients undergoing CRS/HIPEC at our hospital were included. Patient information and anaesthesia-related indicators were collected from the electronic medical record (EMR) system. All subjects were divided into the following two groups: the G group (general anaesthesia) and the GR group (RSB combined with general anaesthesia). Patients in the GR group received 0.375% ropivacaine for BRSB before surgery. The primary outcomes included the total amount of remifentanil and rocuronium, the total consumption of dezocine after surgery, the visual analogue scale (VAS) score and the patient-controlled intravenous analgesia (PCIA) input dose at 1 h (T6), 6 h (T7), 12 h (T8), 24 h (T9) and 48 h (T10) after surgery. Other outcomes were also recorded, such as patient demographic data, the intraoperative heart rate (HR) and mean arterial pressure (MAP), and postoperative complications.

**Results:**

Compared with the G group, the GR group showed a shorter time to tracheal extubation (*P* < 0.05), a decreased total amount of remifentanil and rocuronium (*P* < 0.05), and a reduced VAS score, PCIA input dose and number of PCIA boluses at 1 h, 6 h and 12 h after surgery (*P* < 0.05). However, at 24 h and 48 h after surgery, there were no differences in the VAS score of pain at rest or during motion between the two groups (*P* > 0.05). Moreover, the incidence of hypertension, emergence agitation, delayed recovery, hypercapnia, and nausea and vomiting was lower in the GR group than in the G group (*P* < 0.05). There were no differences in the changes in MAP and HR during the surgery between the two groups (*P* > 0.05). No complications associated with nerve block occurred.

**Conclusion:**

BRSB could provide short-term postoperative analgesia, reduce perioperative opioid consumption and reduce the incidence of postoperative complications. It is an effective and safe procedure in CRS/HIPEC.

## Background

Radical cytoreductive surgery (CRS) combined with hyperthermic intraperitoneal chemotherapy (HIPEC) is considered a standard for the treatment of peritoneal cancer, such as rectal cancer, ovarian cancer, peritoneal pseudomyxoma, and peritoneal mesothelioma [1]. This technique could prolong the long-term survival of patients with a decreased recurrence rate [2]. Although the positive results of this treatment have been proven in previous studies [3–5], because of the large peritoneal surface area involved in this kind of surgery, CRS/HIPEC is time consuming and complex [6], which presents a great challenge for the anaesthesiologist in terms of perioperative management.

Due to the stable respiratory and circulatory support, general anaesthesia is the preferred choice in this surgery. However, long periods of general anaesthesia lead to drug accumulation in the body, followed by increased anaesthesia-related complications, including delayed recovery, respiratory inhibition and cognitive dysfunction [7]. Consequently, exploring better anaesthesia methods for this surgery is still a major concern.

A new approach called ultrasound-guided bilateral rectus sheath block (BRSB) has been proven to ameliorate postoperative pain and reduce the consumption of morphine [8–10]. Nonetheless, there have been no reports on the application of general anaesthesia combined with BRSB in patients undergoing CRS/HIPEC. Based on the information presented above, this retrospective, observational study was conducted to examine the efficacy and safety of BRSB in patients treated with CRS and HIPEC.

## Methods

### Subjects

All patients who underwent CRS and HIPEC at Beijing Shijitan Hospital between August 2016 and December 2018 were retrieved from the institutional database. In this study, the exclusion criteria were as follows: 1. laparoscopic surgery with CRS/HIPEC; 2. intraoperative blood loss volume greater than 1000 ml; 3. mechanic ventilation required after surgery; and 4. use of analgesic techniques apart from BRSB and general anaesthesia. According to this standard, a total of 178 patients were included and divided into the following groups: general anaesthesia (G group, *n* = 89) and general anaesthesia combined with posterior RSB (GR group, *n* = 89).

### Anaesthesia method

**Fig. 1:**
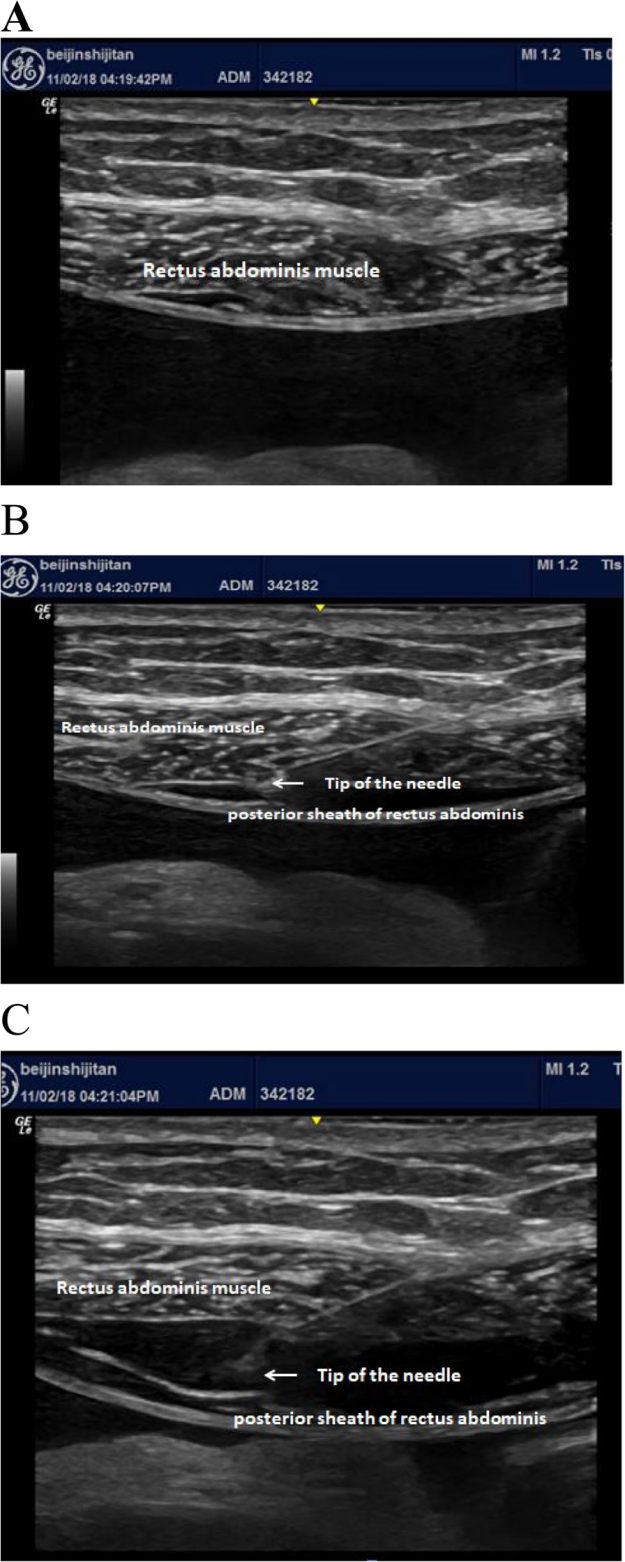
Ultrasound-guided BRSB

General anaesthesia was consistently induced in all patients with intravenous propofol (2 mg/kg), sufentanil (0.5 μg/kg) and rocuronium (0.6 mg/kg). Invasive arterial pressure and central venous pressure were monitored by radial artery puncture (FloTrac/Vigileo®, Edwards Lifesciences, Irvine, CA, USA) and internal jugular vein puncture, respectively, after anaesthesia induction. Anaesthesia was maintained with sevoflurane and remifentanil (effect site target concentration: 4–5 ng/ml), keeping the bispectral index (BIS) between 45 and 55. Rocuronium 0.15 mg/kg was intermittently used to maintain muscle relaxation. In the GR group, before anaesthesia induction, patients received BRSB under ultrasound guidance. The puncture site was placed at the outer edge of the bilateral rectus abdominis at the level of the umbilicus (Fig. 1 a). A total of 0.375% ropivacaine (20 ml) was injected into each side. The spindle-shaped spread of ropivacaine was observed between the posterior sheath of the rectus abdominis and the rectus abdominis itself, implying success of the procedure (Fig. 1 b, c). Patient-controlled intravenous analgesia (PCIA) was applied in both groups after the surgery. Sufentanil 2 μg/kg + palonosetron hydrochloride 0.25 mg was diluted to 100 ml; the background dose was 2 ml/h, and a single dose was 1 ml/h with a 15-min lock-out interval. After the surgery, all patients were sent to the surgical intensive care unit (SICU). If the visual analogue scale (VAS) score at rest after surgery was ≥4, dezocine 5 mg was used as a rescue analgesic.

### Data collection

All the indicators we needed were obtained from the EMR system. The records included patient demographic data, patient medical history, American Society of Anesthesiologists (ASA) grade, and New York Heart Association (NYHA) grade. The HR and MAP were recorded at the time before BRSB (T1), the time of anaesthesia (T2), the time of skin incision (T3), the time of peritoneal thermochemotherapy (T4), and the end of surgery (T5). In addition, the duration of the surgery, time to tracheal extubation (the time after skin closure), total amount of remifentanil and muscle relaxants, total fluid volume, urine volume, and the total volume of allogeneic erythrocytes and plasma infused during the surgery were all recorded. Moreover, after surgery, the occurrence of hypertension (the systolic blood pressure dropped by more than 30% of baseline blood pressure before anesthesia or the SBP < 80 mmHg during surgery), nausea and vomiting, hypoxemia (SpO2 < 90% or PaO2 < 60 mmHg), hypercapnia (PaCO2 > 45 mmHg) and emergence agitation during the recovery period were recorded. The recovery period was considered as the time from switching off inhalation anaesthetics, remifentanil and muscle relaxant to recovery of the patients’ abilities to command movement, orientation, as well as conscious state. When the recovery period of patients is beyond 60 min, it was considered as delayed recovery. The VAS score for pain at rest and during motion, the PCIA input dose and the number of boluses at 1 h (T6), 6 h (T7), 12 h (T8), 24 h (T9) and 48 h (T10) after surgery, as well as the dose of dezocine used as a rescue analgesic, were also recorded. In addition, BRSB-related complications, such as peritoneal puncture, internal organ injury and systemic toxicity, were all recorded.

**Fig. 2:**
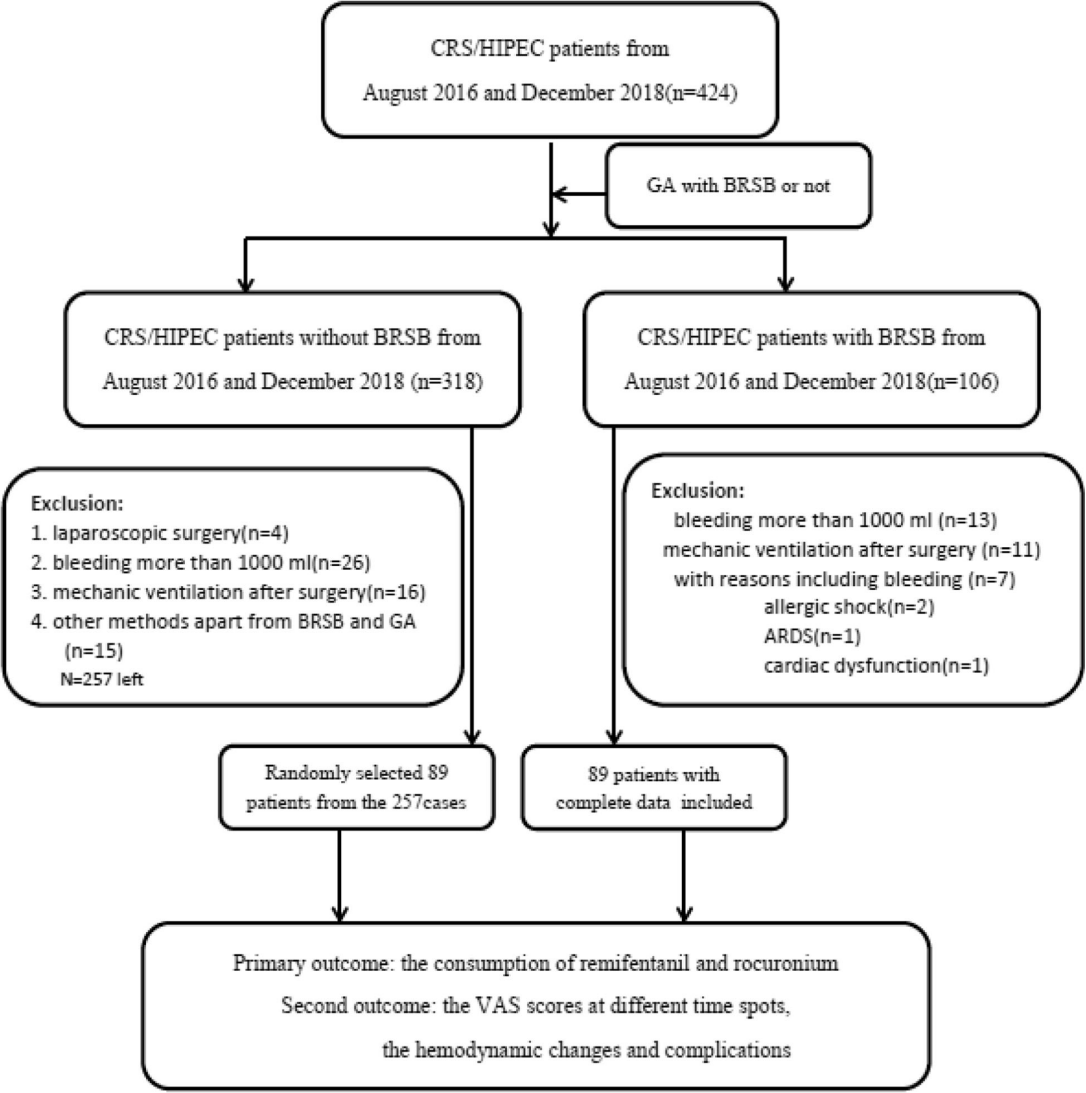
Flow chart showing patient consecutive enrolment and analysis. Abbreviations: CRS/HIPEC, cytoreductive surgery and hyperthermic intraperitoneal chemotherapy; GA, general anesthesia; BRSB, bilateral rectus sheath block; ARDS, acute respiratory distress syndrome; VAS, visual analogue scale

A total of 424 patients underwent cytoreductive surgery combined with hyperthermic intraperitoneal chemotherapy (CRS/HIPEC) during the year from 2016 to 2018 in our hospital. One hundred and six patients received BRSB. Seventeen patients were excluded because of the incomplete data, including 13 patients undergoing intraoperative haemorrhage (blood> 1000 ml), and 4 other patients received mechanic ventilation because of acute respiratory distress syndrome (ARDS), allergic shock, and cardiac insufficiency. Thus, 89 patients with BRSB were eventually obtained. Finally, 89 patients without BRSB were randomly selected to analysis in this study. (Fig. 2).

### Statistical analysis

SPSS 19.0 software was used for statistical analysis. Normal distribution data were recorded as the mean ± standard deviation (SD) and analysed by independent-samples t test for comparison between the two groups. Non-normally distributed data are presented as the median (range) and were analysed by Kruskal-Wallis test. Chi-squared test or Fisher’s exact test was used for categorical data. A *P* value of < 0.05 was considered statistically significant.

## Results

### Characteristics of study population

In total, 178 patients were included in the study. The baseline demographic and surgical variables of patients are presented in Table 1. There were no significant differences in age, sex, body mass index (BMI), basic diseases, ASA grade, NYHA grade, total surgery time, total fluid volume, urine volume, total volume of allogeneic erythrocyte infusion or total volume of plasma (*P* > 0.05). However, the time to tracheal extubation was shorter in the GR group than in the G group (*P* < 0.05). The total amount of both remifentanil and rocuronium used was less in the GR group than in the G group (*P* < 0.05). Thus, posterior RSB could reduce the use of remifentanil and rocuronium during surgery.

**Table 1:** Demographic and surgical variables, mean ± SD

	G group (*n* = 89)	GR group (*n* = 89)	*P* value
Age (y)	46.1 ± 8.2	45.3 ± 8.0	0.512
Sex (male/female)	45/44	36/53	0.176
BMI (kg/m^2^)	24.92 ± 2.40	24.43 ± 1.77	0.123
Medical history			
Diabetes mellitus (n, yes/no)	27/62	25/64	0.742
Hypertension (n, yes/no)	40/49	48/41	0.230
Coronary heart disease (n, yes/no)	11/78	8/81	0.466
ASA grade I/II/III	32/50/7	36/43/10	0.524
NYHA grade I/II	59/30	50/39	0.166
Total surgery time (min)	517.58 ± 87.90	522.97 ± 95.73	0.697
Time to tracheal extubation (min)	49.48 ± 5.07	37.45 ± 3.89	< 0.001
Remifentanil (mg)	4.21 ± 0.50	2.18 ± 0.20	< 0.001
Rocuronium (mg)	101.97 ± 10.85	84.26 ± 9.99	< 0.001
Total fluid volume (ml)	5491.36 ± 587.99	5543.36 ± 541.21	0.541
Urine volume (ml)	1310.56 ± 161.35	1324.38 ± 153.75	0.559
Total volume of allogeneic erythrocyte infusion (ml)	319.55 ± 235.82	329.21 ± 243.18	0.788
Total volume of plasma (ml)	987.64 ± 114.14	973.71 ± 122.99	0.434

*ASA* American Society of Anesthesiologists; *BMI* Body mass index (calculated as weight in kilograms divided by height in metres squared); *NYHA* New York Heart Association; *G* General anaesthesia; *GR* Bilateral rectus sheath block combined with general anaesthesia. Before bilateral rectus sheath block (T1), the time of anaesthesia (T2), the time of skin incision (T3), the time of peritoneal thermochemotherapy (T4), and the end of surgery (T5)

### Changes in Haemodynamic parameters

The changes in HR and MAP are presented in Table 2. There were no significant differences in HR or MAP at any point in time (T1 to T5) between the two groups (*P* > 0.05). The results suggest that BRSB did not affect the haemodynamics of the patient undergoing CRS/HIPEC.

**Table 2:** Haemodynamic parameters in both groups, mean ± SD

Index	Time point	G	GR	*P* value
		(*N* = 89)	(*N* = 43)	
(mmHg)	T1	81.44 ± 12.03	83.74 ± 12.1	0.205
	T2	76.54 ± 7.84	77.91 ± 8.29	0.259
	T3	75.94 ± 7.89	75.99 ± 8.72	0.971
	T4	81.05 ± 9.02	80.82 ± 9.89	0.874
	T5	83.66 ± 8.11	84.19 ± 8.73	0.676
HR (bpm)	T1	73.66 ± 6.36	72.47 ± 7.80	0.266
	T2	76.72 ± 6.24	76.29 ± 7.39	0.678
	T3	78.42 ± 8.79	79.93 ± 7.54	0.218
	T4	78.17 ± 8.25	79.00 ± 10.66	0.561
	T5	84.74 ± 6.19	83.91 ± 6.28	0.368

*MAP* Mean arterial pressure; *HR* Heart rate; *G* General anaesthesia; *GR* Bilateral rectus sheath block combined with general anaesthesia

### Pain-related indicators

Table 3 shows the postoperative VAS score, the PCA input dose and the number of PCA boluses at 1 h (T6), 6 h (T7), 12 h (T8), 24 h (T9) and 48 h (T10) after surgery, as well as the dose of dezocine used as a rescue analgesic. From T6 to T8, compared with the G group, the GR group showed significantly decreased VAS scores of pain at rest and during motion (*P* < 0.05). However, at 24 h and 48 h after surgery, there were no significant differences in the VAS scores of pain at rest and during motion between the two groups (*P* > 0.05). From T6 to T10, the PCIA input dose and the number of PCA boluses were also obviously reduced in the GR group compared with the G group (*P* < 0.05). In addition, as a rescue analgesic, the dose of dezocine after surgery in the GR group was significantly lower than that in the G group (*P* < 0.05).

**Table 3:** Pain-related indicators in both groups, median [range]

	G	GR	*P* value
	(*N* = 89)	(*N* = 89)	
VAS score of pain at rest, [median (range)]			
T6	6 [3, 9]	4 [3, 5]	< 0.001
T7	5 [3, 9]	4 [3, 5]	< 0.001
T8	3 [3, 6]	3 [1, 5]	0.001
T9	2 [1, 3]	2 [1, 4]	0.765
T10	2 [1, 3]	2 [1, 3]	0.314
VAS score of pain during motion [median (range)]			
T6	7 [4, 9]	5 [3, 8]	< 0.001
T7	6 [4, 9]	4 [3, 8]	< 0.001
T8	4 [3, 9]	4 [2, 6]	< 0.001
T9	3 [1, 4]	3 [1, 5]	0.922
T10	3 [1, 4]	3 [1, 5]	0.557
Total infused dose of PCIA [ml, median (range)]			
T6	4 [2, 7]	2 [2, 4]	< 0.001
T7	15 [12, 19]	12 [10, 17]	< 0.001
T8	26 [24, 30]	25 [24, 27]	< 0.001
T9	50 [48, 54]	50 [48, 54]	0.024
T10	100 [96, 100]	97 [96, 100]	< 0.001
Cumulative number of PCIA boluses [median (range)]			
T6	1 [1, 2]	0 [0, 1]	< 0.001
T7	5 [1, 8]	1 [1, 2]	< 0.001
T8	9 [7, 13]	5 [3, 8]	< 0.001
T9	9 [8, 15]	8 [5, 15]	< 0.001
T10	12 [7, 17]	12 [7, 17]	0.047
Total dose of dezocine as a rescue analgesic (mg)	6.07 ± 5.08	15.90 ± 4.10	< 0.001

*VAS* Visual analogue scale; *PCIA* Patient-controlled intravenous analgesia; *G* General anaesthesia; *GR* Bilateral rectus sheath block combined with general anaesthesia. The time at 1 h after surgery (T6); 6 h after surgery (T7); 12 h after surgery (T8); 24 h after surgery (T9); and 48 h after surgery (T10)

### Postoperative adverse events

Adverse events that occurred in the SICU are presented in Table 4. After surgery, there were 29 cases with hypertension, 9 cases of emergence agitation, 7 cases of delayed recovery, 7 cases of hypercapnia, and 12 cases of nausea and vomiting in the G group; fewer cases of all of these events occurred in the GR group (*P* < 0.05). There were no differences in the incidence of hypoxemia between the two groups (*P* > 0.05). There were no complications associated with nerve block in either group.

**Table 4:** Postoperative adverse events in both groups

Adverse events (n, %)	G	GR	*P* value
	(*N* = 89)	(*N* = 43)	
Hypertension	29 (32.58%)	11 (12.36%)	0.001
Emergence agitation	9 (10.11%)	2 (2.24%)	0.029
Delayed recovery	7 (7.87%)	1 (1.12%)	0.030
Hypoxemia	5 (6.74%)	2 (2.24%)	0.254
Hypercapnia	7 (7.87%)	1 (1.12%)	0.030
Nausea and vomiting	12 (13.48%)	4 (4.49%)	0.036
Peritoneal puncture	0 (0%)	0 (0%)	–
Internal organ injury	0 (0%)	0 (0%)	–
Systemic toxicity	0 (0%)	0 (0%)	–

*G* general anaesthesia; *GR* Bilateral rectus sheath block combined with general anaesthesia

## Discussion

In this retrospective study, we examined the efficacy and safety of BRSB combined with general anaesthesia in patients undergoing CRS/HIPEC. Regarding efficacy, the results show that ultrasound-guided BRSB significantly reduced the total dose of remifentanil used during the surgery and shortened the time to tracheal catheter extraction, which is consistent with the findings of previous studies of other surgeries [11, 12]. In addition, RSB reduced the total dose of rocuronium in this study, which may be associated with the high concentration of ropivacaine used in the study.

RSB also effectively relieved postoperative pain. In this study, we found that the VAS scores of pain at rest and during motion were all lower in the GR group than in the G group at 12 h after surgery. However, at 24 h and 48 h after surgery, there were no differences in the VAS scores of pain at rest and during motion between the two groups, suggesting that the analgesic effects of a single BRSB remained within 12 h after surgery. This result may be different from the findings of others [13]. Cho et al. reported that at 12 h after surgery, there were no differences in the VAS scores of pain at rest and during motion between the RSB and non-RSB groups. The discrepant results may be related to differences in the concentration of ropivacaine and the physical constitution of patients. A high concentration can prolong the duration of action of a local anaesthetic [14]. In this study, we selected 0.375%, not 0.25%, ropivacaine. Additionally, these patients undergoing CRS/HIPEC may have been adaptive to pain. Furthermore, compared with the control group, the RSB group showed a reduced total infused dose of sufentanil as PCIA, number of PCA boluses within 48 h after surgery and total dose of dezocine used as a rescue analgesic after surgery. These results further prove the role of RSB in providing short-term postoperative analgesia.

We also examined the safety of RSB. During the surgery, ultrasound-guided BRSB had no significant effects on the haemodynamics of patients during surgery compared with general anaesthesia alone. In terms of postoperative adverse events, the results show that compared with the control group, the RSB group showed a reduced incidence of hypertension, emergence agitation, delayed recovery, hypercapnia, and nausea and vomiting, which might be correlated with the decreased analgesic and muscle relaxant doses. No RSB-related complications occurred in any patient. These data indicate that RSB could reduce the risk of complications associated with general anaesthesia and is safe for patients.

RSB, an established technique, has regained popularity in clinical applications [15–17]. Previous studies have demonstrated that this technique could achieve relaxation of the anterior abdominal wall [8, 16]. Bashandy reported that anterior branches of the T7-T12 thoracic nerve and the L1 lumbar nerve travelled through the plane of the transverse abdominis muscle, entered the rectus abdominis sheath, and distributed on the surface of the skin [18]. The main process of RSB is to inject local anaesthetics between the rectus abdominis and the posterior sheath of the rectus abdominis [19]. Therefore, RSB exerted a good effect in terms of perioperative analgesia for median abdominal incisions [20]. A midline incision is required in this kind of surgery. Thus, based on these results, RSB could meet the need for analgesia in these patients.

In addition, for a long time, epidural analgesia (EA) was thought to be an effective method for abdominal surgery [21–23]. Studies have proved that epidural analgesia could maintain a good analgesic effect and reduce perioperative opioid consumption, including in this type of surgery [21, 24]. However, the safety of EA in CRS/HIPEC remains controversial, especially regarding effects on coagulation and circulatory function. Coagulation dysfunction and profound fluid loss are the main characteristics of patients with peritoneal cancer [25, 26], which might limit the administration of EA.

Although epidural catheter is standard of care in Solanki’s guideline [27]. In our hospital, epidural catheter in not the standard of care. We performed general anesthesia combined with epidural anesthesia in some patients to reduce the consumption of intravenous drugs and provide perfect analgesia. But coagulation dysfunction and profound fluid loss are the main characteristics of patients with peritoneal cancer. In our previous study, we found that the mean arterial pressure of patients undergoing epidural anesthesia was difficult to be maintained in the surgery. Besides, there were many patients with coagulation dysfunction before surgery, who were not suitable for the epidural anesthesia. These results we found in clinical were similar with others’ researches. Kajdi and colleagues reported a case of epidural haematoma in their study [26]. Godden found that the incidence of hypotension in the EA group was obviously higher than that in the RSB group [28]. Consequently, RSB could be a better choice than EA in CRS/HIPEC.

Additionally, there are some limitations to this study. First, all the data of this study were collected from the EMR system. As this was a retrospective study, the findings are not as persuasive as those of a randomized, controlled study. We plan to conduct prospective studies to explore the comprehensive influence of RSB in this surgery. Second, we only examined the application of BRSB established with a single injection, which provides only a short-term analgesic effect. The efficacy of continuous analgesia with BRS catheters in CRS/HIPEC remains unclear and needs further exploration. Third, in our article, the results are initially presented according to the different aspects. The primary outcome of this study is the total consumption of remifentanil during the surgery. Other indicators were belonged to second outcomes.

## Conclusions

In conclusion, BRSB could provide good postoperative analgesia, reduce perioperative opioid consumption and reduce the incidence of postoperative complications. This is an easily applicable and safe procedure in CRS/HIPEC.

## Data Availability

The datasets used and/or analyzed during the current study are available from the corresponding author on reasonable request.

## Abbreviations

RSB: Rectus sheath block
BRSB: Bilateral rectus sheath block
CRS: Cytoreductive surgery
HIPEC: Hyperthermic intraperitoneal chemotherapy
EMR: Electronic medical record
VAS: Visual analogue scale
PCIA: Patient-controlled intravenous analgesia
HR: Heart rate
MAP: Mean arterial pressure
BIS: Bispectral index
SICU: Surgical intensive care unit
ASA: American society of anesthesiologists
NYHA: New York heart association
SpO2: Pulse oximetry
PaCO2: Partial pressure of carbon dioxide
PaO2: Oxygen partial pressure
BMI: Body mass index
